# Palmoplantar Psoriasis: A Clinico-Pathologic Study on a Series of 21 Cases with Emphasis on Differential Diagnosis

**DOI:** 10.3390/diagnostics12123071

**Published:** 2022-12-06

**Authors:** Giuseppe Broggi, Maria Failla, Andrea Palicelli, Magda Zanelli, Rosario Caltabiano

**Affiliations:** 1 Department of Medical and Surgical Sciences and Advanced Technologies “G.F. Ingrassia”, Anatomic Pathology, University of Catania, 95123 Catania, Italy; 2 Pathology Unit, Azienda USL-IRCCS di Reggio Emilia, 42123 Reggio Emilia, Italy

**Keywords:** palmoplantar psoriasis, diagnosis, histopathology, differential diagnosis, case series

## Abstract

Palmoplantar psoriasis (PP) is a relatively uncommon variant of psoriasis that affects palms and soles, and that frequently shares both clinical and histologic features with chronic eczema, hyperkeratotic hand dermatitis and allergic contact dermatitis. The present study aims to characterize the histologic features of PP on a series of 21 cases. The following morphological features and their distribution were included: parakeratosis, dilated vessels in papillary dermis, psoriasiform acanthosis with elongation of rete ridges, perivascular lymphocytic infiltrate, decrease/loss of granular layer, Munro’s microabscesses, spongiform pustules of Kogoj, spongiosis and lymphocytic exocytosis. The main diagnostic clues and histologic differential diagnoses are also discussed.

## 1. Introduction

Psoriasis is a chronic inflammatory skin disease that affects 2% to 5% of the general population and is typically characterized by keratinized plaques with white scales [1,2,3]. Psoriasis patients have individual disease expression, with different degrees and severity of skin involvement [4].

Palmoplantar psoriasis (PP) is a variant of psoriasis that specifically affects the skin of the palms and soles of individuals of all ages. It represents 3–4% of all psoriasis cases [5,6,7]. PP is often classified into subtypes based on the morphology of the lesions: it may manifest with thick hyperkeratotic plaques, sterile pustules, or mixed morphology [4,7]. The thick hyperkeratotic plaque, which is the most common subtype, manifests with sharply outlined, erythematous, scaly plaques with hyperkeratosis and the absence of pustular lesions, whereas the pustular variety includes macroscopic, sterile pustules and erythema, with yellow-brown spots [4].

Lesions are commonly symmetrically distributed. Sites other than the hands and feet are frequently involved, with studies showing 33% of patients having up to 10% of their body surface area (BSA) involved [4].

Even though it is quite uncommon, this disease needs to be correctly diagnosed, since it may cause a great impairment in quality of life. Clinically, patients experience itching, soreness, burning, pain and fissuring that can cause marked physical discomfort and functional disability [7]; although involvement of the palms and soles often affects <5% of total body surface area with a low PASI score, these patients may suffer from greater physical limitations than individuals with psoriasis in other areas [8,9].

Furthermore, this variant is generally considered recalcitrant to treatment, even though new biologic therapies have shown promising results for its management [8,9].

Palmoplantar psoriasis is considered to be caused by a combination of genetic and environmental factors. The most common genetic factor associated with PP includes the human leukocyte antigen (HLA) Cw6, whereas environmental triggers include smoking, specific detergents, seasonal changes, friction, and repetitive trauma of the area, which may cause a relapse of the disease [8,9]. Dobrică et al. delineated a significant alteration of the redox balance, with a significant decrease in antioxidant enzymes and antioxidant markers, and an increase in pro-oxidant molecules in psoriasis [10].

The clinical features of PP frequently overlap with chronic eczema and the frequent co-occurrence of these two conditions could lead to a misdiagnosis [4,11,12]. Both diseases share some pathological features, such as epidermal hyperplasia, parakeratosis and spongiosis, but since these two entities need different treatments, proper identification is essential for a successful outcome [4,11,12].

The differential diagnosis of palmoplantar psoriasis includes contact dermatitis, pityriasis rubra pilaris, acquired palmoplantar keratoderma, and tinea pedis/manuum. Other dermatoses, such as palmoplantar pustulosis and acrodermatitis continua of Hallopeau, are generally included in the spectrum of disorders classified as palmoplantar psoriasis [8].

A histopathologic examination of psoriatic lesions usually reveals moderate to strong parakeratosis, regular acanthosis, decrease/loss of the granular layer, thinning of suprapapillary plates, edema of papillary dermis, Munro’s microabscesses (neutrophils in the stratum corneum) and/or spongiform pustules of Kogoj [2,3]. In addition, foci of parakeratosis, vertically oriented, alternating with orthohyperkeratosis, are often seen [13].

The aim of this study is to characterize the specific histopathologic features of palmoplantar psoriasis, in order to better understand this entity, to improve its management and to provide pathologists with a practical and useful diagnostic approach for dealing with these challenging lesions. Differential diagnostic clues are also emphasized.

## 2. Materials and Methods

Although the present research complied with the Helsinki Declaration, the non-interventional retrospective nature of our study did not require any informed consent by the local research ethics committee.

We performed a retrospective study on all PP cases retrieved from our database, from January 2017 to July 2022.

The following inclusion criteria were adopted: (i) no detected allergies, confirmed by a negative patch test, and (ii) no previous topical or systemic treatment for at least 1 month. Cases that presented pustular lesions were tested for bacterial infections and could only be included if the microbiologic culture swab of the lesions was negative. Cases with suspicion/evidence of other papulo-squamous conditions by clinical examination and/or histologic findings, including acrodermatitis continua of Hallopeau, were excluded.

All hematoxylin and eosin (H&E)-stained slides were evaluated by two pathologists (M.F. and R.C.) in order to identify features typical of classic psoriasis, such as psoriasiform acanthosis, parakeratosis, Munro’s microabscesses, spongiform micropustules of Kogoj, dilated and/or tortuous papillary dermal vessels, perivascular lymphocytic infiltrate, and the reduction/absence of the granular layer of the epidermis. We also investigated other histologic features, including spongiosis, lymphocytic exocytosis, and papillary dermis edema, which is not commonly present in psoriasis, but frequent in other skin conditions such as eczema [14].

Features such as spongiosis, lymphocytic exocytosis, congest/dilated vessels in papillary dermis, perivascular lymphocytic infiltrate and psoriasiform acanthosis, were rated on a scale from 0 (absent), 1 (focal), 2 (diffuse/moderate), to 3 (strikingly prominent/abundant).

## 3. Results

A total of 21 patients met the inclusion criteria: 12 (57.14%) males, 9 (42.86%) females, ranging from 22 to 71 years of age (mean age: 47.76 years). Table 1 summarizes the histologic features found in our series.

**Figure 1:**
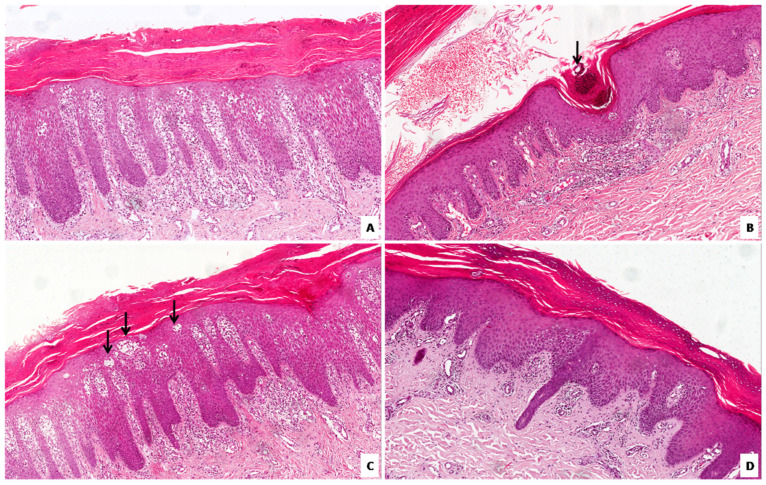
Histopathology of palmoplantar psoriasis. (**A**) Hyperparakeratosis associated with psoriasiform acanthosis with thinning of the suprapapillary plate and partial loss of the granular layer is seen; dilated vessels and moderate perivascular lymphocytic infiltrate are also shown at the papillary dermis. (**B**) Medium magnification showing a case of palmoplantar psoriasis with a Munro’s microabscess in the stratum corneum (arrow). (**C**) Marked spongiosis along with spongiform pustules of Kogoj (arrows) is seen. (**D**) Medium magnification showing spongiotic features associated with lymphocytic exocytosis in palmoplantar psoriasis. (Original magnifications: (**A**–**D**), 200×).

All 21 cases exhibited parakeratosis and parakeratotic areas, that alternated both vertically and horizontally with orthokeratotic areas (Figure 1A). The granular layer was normal in 2 biopsies (9.5%), decreased or focally lost in the remaining 19 (90.5%) (Figure 1A). It is worth noting that the loss of the granular layer was always observed in correspondence with the hyperkeratotic areas.

All cases had congested and dilated vessels in the papillary dermis (Figure 1A). In 3 cases (14,2%) this finding was focal, in 12 (57.1%) cases it was more diffuse/moderate, whereas in 6 (28.5%) cases it was strikingly prominent. Psoriasiform acanthosis of the epidermis was observed in 17 (80.9%) cases (Figure 1A). In 14 (66.6%) of these cases, it was particularly evident with a marked elongation of rete ridges, while in the remaining 3 (142%) cases it was focal.

All biopsies showed various levels of perivascular lymphocytic infiltrate (Figure 1A), which was scarce and focal in 2 (9.5%) cases, moderate in 5 (23.8%) cases, and diffuse and abundant in the remaining 14 (66.6%) cases.

Munro’s microabscesses, a typical feature of classic plaque psoriasis, were a common finding (Figure 1B). Only 2 (9.5%) cases did not exhibit them, whereas they were present in the remaining 19 (90.5%) cases. On the other hand, spongiform pustules of Kogoj were found in only 3 (14.2%) biopsies (Figure 1C).

Spongiosis, typical of eczema, was absent in only 2 (9.5%) cases, focal in 11 (52.4%) cases, moderate in 4 (19.0%) cases and marked in the remaining 4 (19.0%) cases. In our evaluation, the level of spongiosis correlated with the level of lymphocytic exocytosis (Figure 1D): areas with normal epidermis had no lymphocytic exocytosis, whereas spongiotic areas always showed lymphocytic exocytosis to some extent, which was greater when spongiosis was more prominent and vice versa.

## 4. Discussion

Palmoplantar psoriasis is a challenge for both dermatologists and pathologists, since it shares many histopathological features with other more common entities that can affect the palms and the soles.

In this study, we analyzed typical histologic features of PP and classic plaque psoriasis, and additional features that are more frequently associated with PP, while being rare in classic plaque psoriasis.

The features we observed more frequently included: parakeratosis alternated with orthokeratotic areas, presence of congested, dilated, and tortuous vessels in the papillary dermis, and decrease/loss of the granular layer. These findings, including neutrophils in the stratum corneum, could be considered a diagnostic clue of PP. Spongiform pustules of Kogoj were observed in only a few cases (14.2%).

Differential diagnosis between PP and hand eczema (HE) or hyperkeratotic hand dermatitis (HHD) is difficult because of their overlapping clinical findings [15]. Histologic clues and their distribution among these entities are summarized in Table 2. HHD is a clinical variant of chronic hand dermatitis, typical of the adult, generally limited to the palms, but often involving the volar surfaces of the fingers. It shares with PP the small foci of parakeratosis, the predominantly perivascular chronic inflammatory cell infiltrate in the papillary dermis, and lymphocytic exocytosis [15]. Park et al. observed the loss of the granular layer more frequently in PP cases rather than HE or HHD cases [15]. They also noted that psoriasiform epidermal hyperplasia is significantly more frequent in PP and HHD than in HE, in which an irregular epidermal hyperplasia is more common [15]. An et al. also observed that confluent parakeratosis, an absent granular layer, and psoriasiform epidermal hyperplasia were more common in palmar psoriasis and HHD, rather than chronic hand dermatitis [16]. Rao et al., comparing PP cases and HHD, concluded that these diseases share many features, such as focal parakeratosis, the presence of neutrophils and fibrin globules in the stratum corneum, hypogranulosis, acanthosis, spongiosis, rete ridge pattern, or vascularity [17]. Confluent parakeratosis, suprapapillary thinning and dermal edema were more frequent in PP biopsies, while an inflammatory infiltrate, confined only to the papillary dermis, was a significant feature in palmoplantar dermatitis [17].

In our study, spongiosis and lymphocytic exocytosis, often considered suggestive for other cutaneous diseases, have both been frequently observed in PP cases and, therefore, these features should not exclude the diagnosis of PP.

The differential diagnosis of PP also includes allergic contact dermatitis (ACD) [5]. In a study by Cesinaro et al., regular epidermal hyperplasia and marked parakeratosis were found to be more frequent in PP than in ACD cases [18].

In agreement with previous studies [15,17,19,20], we also observed multiple parakeratotic foci alternating with ortho-hyperkeratosis, regular epidermal hyperplasia and marked parakeratosis, more frequently in PP than in ACD. On the other hand, irregular epidermal hyperplasia and a higher number of S100 protein-positive dendritic cells favor the diagnosis of ACD. Moreover, eosinophils, dilated, and tortuous capillaries in the dermis, common features in ACD, are not typically seen in PP lesions. Aydin et al. focused on acute-onset PP, which often showed spongiosis generally restricted to the lower epidermis, as a confounding factor [19]. In these cases, initial biopsies show, in addition to intense spongiosis, some vesiculation, mounds of parakeratosis containing neutrophils, dilated vessels in the papillary dermis, and a mild, superficial perivascular infiltrate of lymphocytes [19]. As we confirmed in our study, in this setting the presence of multiple parakeratotic foci alternating with ortho-hyperkeratosis favored a diagnosis of PP.

**Table 2:** Histologic differential diagnosis [15,17,20,21].

Histologic Features	PP	HE	HHD	ACD
Small foci of parakeratosis	~100%	~24%	~72%	~48%
Chronic inflammatory infiltrate in papillary dermis	~100%	~62%	~75%	~36%
Loss of the granular layer	~91%	~24%	~28%	~0%
Psoriasiform epidermal hyperplasia	~81%	~37%	~72%	~0%
Irregular epidermal hyperplasia	~9%	~63%	~28%	~100%
Spongiosis	~91%	~27%	~24%	~100%
Lymphocytic exocytosis	~91%	~83%	~100%	~77%
Eosinophils in the dermis	~0%	~10%	~0%	~84%
Neutrophils in the stratum corneum	~91%	~15%	~12%	~0%

Abbreviations: PP, palmoplantar psoriasis; HE, hand eczema; HHD, hyperkeratotic hand dermatitis; ACD, allergic contact dermatitis.

A biopsy is generally not necessary when the clinical manifestations are typical [5]. Histopathological examination is the main diagnostic tool in difficult cases of PP, but it is unquestionable that, especially in these cases, pathologists should work alongside dermatologists who should always perform the biopsy at the right time and at the right site. As the biopsy reflects the clinical image, it should be performed before any treatment, when lesions are clearly visible.

Regarding the selection of biopsy areas in cases of PP, the hand should be preferred, considering that the lower limbs and feet may show some confounding factors, such as serum in the stratum corneum. Some clinicians, by investigating the specific features of psoriasis of the leg, showed that, in this area, psoriasis biopsy samples were significantly less likely to show typical histological criteria, such as regular hyperplasia, suprapapillary thinning, and “kissing vessels”, whereas the most valuable criteria to distinguish psoriasis on the legs from stasis dermatitis were the presence of neutrophils in the cornified layer and staggered parakeratosis [21].

The aim of our study was to investigate novel potential diagnostic clues and peculiar histologic features of PP, which previous studies might have overlooked. Although we are aware that the histopathologic features of this entity have been already described, we would like to emphasize that there are still many features overlapping with those of other entities that pathologists must include in the differential diagnosis, including HE, HHD, and ACD. Accordingly, the additional aim of this study was to provide a practical diagnostic guide useful when dealing with these challenging lesions.

In summary, according to our findings, the features that suggest a PP diagnosis the most are parakeratosis alternated with orthokeratotic areas, loss of the granular layer, and neutrophils in the stratum corneum, while spongiosis and lymphocytic exocytosis do not exclude the diagnosis. A limitation to our study is the relatively small sample size, therefore we suggest and encourage further studies to investigate the histopathologic features of palmoplantar psoriasis.

## Figures and Tables

**Table 1:** Histologic features from our series.

Histologic Features	Absent (0)	Present	Focal (1)	Moderate (2)	Marked (3)
Parakeratosis	0	/	9 (42.86%)	12 (57.14%)	
Vertically oriented parakeratosis alternated with orthokeratosis	0	21 (100%)	/	/	/
Congest and dilated vessels in papillary dermis	0	/	3 (14.2%)	12 (57.14%)	6 (28.5%)
Psoriasiform acanthosis	4 (19.0%)	17 (80.9%)	/	/	/
Elongation of rete ridges	0	/	3 (14.2%)	14 (66.6%)	/
Perivascular lymphocytic infiltrate	0	/	2 (9.5%)	5 (23.8%)	14 (66.6%)
Decrease/loss of the granular layer	2 (9.5%)	19 (90.5%)	/	/	/
Munro’s microabscesses	2 (9.5%)	19 (90.5%)	/	/	/
Spongiform pustules of Kogoj	18 (85.8%)	3 (14.2%)	/	/	/
Edema in the papillary dermis	14 (66.6%)	/	5 (23.9%)	2 (9.5%)	/
Spongiosis	2 (9.5%)	/	11 (52.4%)	4 (19.0%)	4 (19.0%)
Lymphocytic exocytosis	2 (9.5%)	/	11 (52.4%)	4 (19.0%)	4 (19.0%)

## Data Availability

All data presented in this study are available from the corresponding author upon reasonable request.
